# Review on Selected Aggression Causes and the Role of Neurocognitive Science in the Diagnosis

**DOI:** 10.3390/ani12030281

**Published:** 2022-01-24

**Authors:** Aleksandra Kleszcz, Paulina Cholewińska, Greta Front, Jakub Pacoń, Robert Bodkowski, Marzena Janczak, Tadeusz Dorobisz

**Affiliations:** 1 Institute of Animal Breeding, Wroclaw University of Environmental and Life Sciences, 51-630 Wroclaw, Poland; 112564@student.upwr.edu.pl (G.F.); robert.bodkowski@upwr.edu.pl (R.B.); marzena.janczak@upwr.edu.pl (M.J.); 2 Department of Genetics, Wroclaw University of Environmental and Life Sciences, 51-630 Wroclaw, Poland; jakub.pacon@upwr.edu.pl; 3 Department of Vascular Surgery, Wroclaw Medical University, 50-556 Wroclaw, Poland; tdorobisz@gmail.com

**Keywords:** dogs, aggression, diet, hormones, neurocognitive science

## Abstract

**Simple Summary:**

Aggression in dogs is often a reason for abandonment and/or euthanasia. Recently, knowledge about aggression has been subjected to more detailed analysis. In recent years, it has been studied in terms of factors affecting it, such as diet (especially nutritional supplements) and physiology (endocrine system). In addition, recently, new methods of brain research, such as neurocognitive research, have appeared, which enable a significant increase in knowledge about dog behavior, including aggression.

**Abstract:**

Aggression as a behavior is not always desirable, often ends in abandonment and/or euthanasia. However, it is possible to prevent the occurrence of unwanted aggression in domestic dogs. Aggression is not a fully understood phenomenon. In recent years, many studies have focused on the influence of diet and physiology (including the endocrine system) on the emergence of behavioral disorders. In particular, the emphasis was put on nutritional additives such as fatty acids, amino acids, and probiotics. In addition, the possibility of using neurocognition in the observation of abnormal behavior in dogs has also been discussed, which may allow for a more detailed determination of the basis of aggressive behavior in dogs. In this review, the concepts related to aggression and its potential causes have been gathered. In addition, the possible influence of diet and hormones on aggression in dogs has been discussed, as well as the application of neurocognition in the possibility of its diagnosis.

## 1. Introduction

It is estimated that dogs are the first animal species domesticated by humans. As a result of selective breeding, about 400 different breeds of dogs are recognized around the world, representing a large variety in terms of size, use, or weight [1,2,3]. In addition, keeping dogs was associated with the need to learn about their behavior in order to maintain proper relations between the dog and the owner, as well as to eliminate the undesirable behavior, such as aggression [1,2,3,4]. 

To aim to generalize the dogs’ behavior and understand their behavioral needs, ethograms have been developed. Communication of any kind, including aggression, is a natural part of a dog’s ethogram. An ethogram is a species-specific list of natural behavior. Dog ethograms include affiliate, agonistic, defensive, sedative, sexual, demonstrative, warning, stressful, playful, grooming, exploratory, related to hunting, or related to the reduction of stress [5,6]. Additionally, in recent years, new methods have been developed to help understand how a dog’s brain works: neurocognition. The combination of the dietary aspect, physiology (endocrine system), and neurocognition may allow for a thorough understanding of the impact of aggression on the dog’s body and to obtain more detailed information on the basis of its occurrence [7,8,9,10].

Understanding the causes of the occurrence of aggression in dogs is crucial to both animal welfare and the safety of their caregivers. In the circumstances in which a dog shows undesirable aggression, they can inform the competent caregivers about the disposition of the animal, as well as take appropriate measures to eliminate aggressive behavior [4]. Additionally, in North America, aggressive behavior is strongly contributed toward the abandonment and/or euthanasia of animals, especially dogs [8]. Many types of aggression can be distinguished in dog behavior depending on motivation (defensive/offensive), target (other animals, owner, unfamiliar people), etc. In addition, the definition of the underlying cause of aggression has not been fully explained, as it can be both behavioral (related to unmet needs, especially in the early time of life, anxiety or stress, poor socialization, etc.), diseases (such as cancer, brain disorders or diet, etc.) or genetics [9,10,11,12,13,14,15]. Recently, apart from the use of appropriate training for behavioral disorders, the diet and physiology (including the endocrine system) of dogs have also come to be seen as an aspect of the disorders [14]. In particular, the focus was placed on nutritional additives such as fatty acids, amino acids, and probiotics. 

In this review, information on the concepts related to aggression and its causes has been gathered. In addition, the possible influence of diet and hormones on aggression in dogs has been described, as well as the application of neurocognition in its diagnosis. The review is based on searches following databases: Pubmed, Web of Science, and Google Scholar databased. Only studies in peer-reviewed scientific journals published in English between January 2000 and May 2020 were included

## 2. Aggression—Definitions, Types, Consequences, and Possible Causes

Dog aggression can be divided depending on the mental state of the dog and the circumstances in which the aggression appears. Acts of aggression can be categorized against dogs, people, other animals, and moving objects, such as bicycles and cars [10,13,15] (Table 1). 

In this paper, however, the definition created by James O’Heare [20] will be used. The author summed up aggression as “*an attack, a deliberate attack, or a threat of an attack on another individual*”.

**Figure 1:**
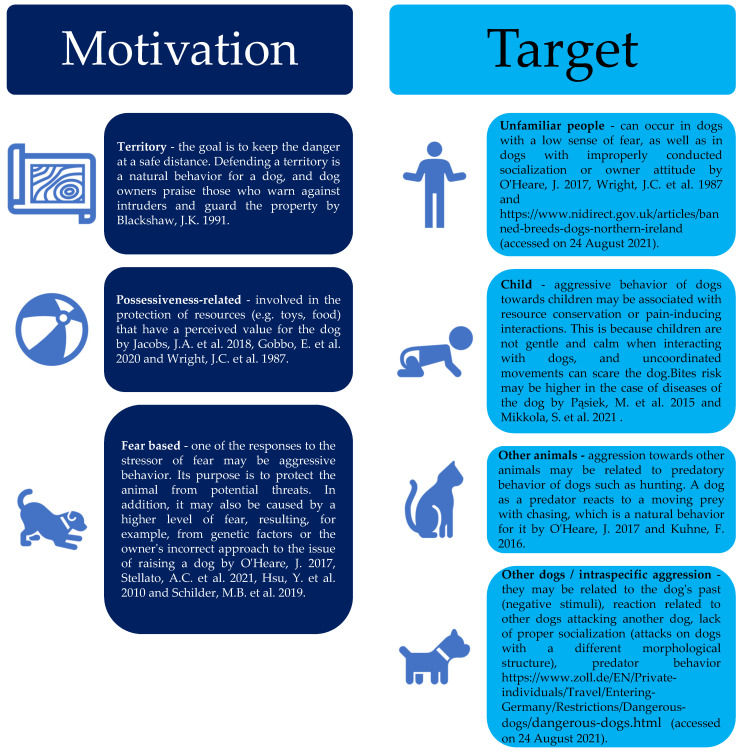
Selected types of aggression in dogs depend on motivation target [9,13,20,23,24,25,26,27,28,29,30,31,32].

The types of dog aggression can be categorized according to the sequence of behaviors and the circumstances in which they occur. The following types of aggression can be distinguished; defensive, distancing, territorial, maternal, irritable, fearful, displaced, competitive between dogs, competitive between dogs and people, possessive, possessive, between dogs belonging to different social groups, hunting, excessive, on command, etc. Figure 1 [17].

Aggression is a natural part of dog behavior, and it is part of the ethogram. However, excessive aggression disrupts the relationship between the dog and its environment [17,18,19,20]. Aggression can be based on motivational basis (territorial-, fear-, possessiveness-related, etc.) or target basis (stranger-, owner-, dog-directed, etc.) [33]. The change of motivation occurs most often when one of the dog’s needs has not been met. For example, in a hungry dog, the change of motivation will manifest itself in looking for food, devoting all the time to it, including the rest period. It will be accompanied by anxiety, distraction, and irritability. The change of motivation also occurs at the time of the onset of the disease. Pain can lead to an avoidance of touch and a closer relationship with the environment and prompts the animal to defend itself, thus showing aggressive behavior [10]. Frustration can change your motivation, which may occur when a reward for expected behavior is missing/delayed or when the dog cannot achieve the goal [34,35]. Frustration-induced behavior can vary depending on the situation and the nature of the dog. As a result, the dog may increase efforts to achieve the desired goal or vocalize [36,37]. Frustration has been found to be an important emotion that influences a number of behavioral problems in dogs. Barrier frustration may arise when the dog is unable to achieve the desired goal by restricting its accessibility, such as with a leash, which may result in diversion and/or aggressive behavior [37,38]. Studies carried out in the years 2008–2016 showed that aggression against unfamiliar dogs (aggression targeted at the dog) had been shown by 22–47% of studied individuals. In many cases of such aggression, the death of one of the dogs involved can be a consequence [39,40]. A study by Van der Borg et al. [41] on Rottweilers showed that as many as a quarter of dogs (mainly without pedigrees) showed behavior related to aggression targeted at both humans and animals. In addition, they were also, in most cases, aggressors in a given interaction. The occurrence of targeted aggression in dogs in such situations may be due to a low level of anxiety (when the aggressor is the dog). Some breeds originally used as protection and guard dogs have a natural predisposition to low levels of anxiety [42,43,44]. On the other hand, when a dog with a high level of fear is subjected to a “stressor” such as unfamiliar people, other pets (such as cats or dogs), or the environment, it may result in defensive aggression [43,44]. Aggression based on fear can also be related to defensive aggression. This aggression is related to the animal’s reaction to a factor that it considers to be a threat, causing fear. This type of factor can also cause physiological and behavioral changes. The main physiological changes are increases in blood pressure, changes in cortisol levels, body temperature, increases in heart rate and blood glucose levels. In terms of behavioral changes, activation takes place at this time of the sympathetic adrenal medullary axis and the hypothalamic-pituitary-adrenal cortex axis [30,45,46,47].

The occurrence of aggression in dogs has consequences related to their abandonment or euthanasia. Additionally, in the case of some breeds, their keeping or breeding has been subject to legal regulations, such as American Pit Bull Terrier or Tosa Inu in most European countries [26,48,49,50]. The created lists of aggressive dog breeds are controversial and do not provide certainty about the possibility/risk of aggressive dog’s behavior. Therefore, the issues of risk assessment are important, primarily based on two main aspects: the first is the ability to predict dog bite risk factors in the general canine population, and the second is how to assess and predict future risk to society [51,52,53]. In a study by Notari et al. [45], it was suggested that aggressive behavior mainly related to early experience, bad socialization process, origins (genetics factor), and welfare of dogs can have play an important role in both intraspecific and interspecific aggression [20,44,54,55,56,57,58].

However, aggressive reactions may occur because of poor health and well-being. Dog aggression can be caused by behavioral and health problems. Failure to meet the basic needs related to the animal’s welfare (access to water, food, play, the possibility of socialization) may cause due to stress, aggressive behavior, as well as health problems [52,53]. In the absence of access to water or food, the dog may search for them in inappropriate places such as toilets or garbage cans. Strengthening inappropriate feeding behaviors (including the speed of food, amount of food given, bowl positioning, etc.) can lead to stomach twists, choking, aggression, fighting for food and defending the bowl, stress, and asking for food [22,57,58,59,60].

When it comes to meeting physiological needs, the dog should be provided with appropriate space. In the studies on dogs staying in the shelter, it was shown that increasing the space twice had a positive effect on the behavior of dogs. Similar results were also obtained by Clark et al. [61] and Hetts et al. [58], which in turn indicates that both genetic and environmental factors can influence a dog’s behavior [39,61,62]. Otherwise, stress may occur due to the need to urinate or defecate or due to anticipating the penalty that awaits the dog for urinating or defecating in the wrong place. By praising the dog for inappropriate behavior or the owner not reacting to such behavior, the dog repeats it. This behavior of the owner be the cause of the reinforcement of inappropriate behavior and lead to the formation of undesired habits and situations resulting from them, e.g., barking at the sound of the doorbell. Improper behavior may be strengthened accidentally and unknowingly, also during conscious, as the guardian might think, training with a clicker. If the dog’s praise occurs too long after the desired behavior, the owner may unwillingly reinforce the following one, thus strengthening unwanted behaviors [63]. Research conducted in recent years also suggests that significantly fewer behavioral problems were shown in dogs that were trained solely through rewards, compared to dogs that were trained using only some form of punishment or a combination of both. The potential negative behavioral effects of aversive training techniques have also been identified in other studies such as Schilder and van der Borg [60], Hiby et al. [22], or Blackwell et al. [39]. During the period of socialization, the dog should get to know and establish positive contact with as many things, situations, and living creatures as possible. Socialization should last a dog’s life. However, it is one of the most important stages in a puppy. Properly socialized puppies are less likely to exhibit undesirable behavior and can develop a positive, lifelong relationship with their owner. Socialization practices should be age-appropriate (start within a few days of birth and should continue into adulthood as previously mentioned). They should be designed to expose the dog in a controlled and enjoyable manner to the many types of experiences, people, and objects it may encounter during its lifetime. Individuals that are properly socialized as puppies are less likely to exhibit behavioral problems in adulthood, including aggression and fear. Otherwise, stress may be manifested in certain situations and coped with, for example, through aggression. Dogs that have undergone a socialization program are much more self-confident and can cope with stressful situations more easily than those that have been less socialized [64,65,66,67,68].

The psychosomatic background, on the other hand, is associated with somatic diseases that have an inhibitory and productive effect on behavior. The somatic diseases affect the previous factors, less intense or not manifested behaviors at all, for example, aggression, defecation, and urination in the wrong places, vocalization, food and water intake, or self-mutilation [10,69]. The occurrence of somatic diseases such as thyroid disease, cancer (especially those related to the nervous system) influences behavior by a general change of motivation. Diseases such as brain cancer can direct disturbance of the brain’s work, which may result in, among others, the occurrence of aggressive behavior. Diet can significantly influence the occurrence of aggressive behavior in dogs too [69,70,71,72,73]. 

## 3. Selected Aspects of Dogs’ Diet and the Occurrence of Aggression

A properly balanced is the basis for the proper functioning of the animal and its health. There are many specialist diets available on the market to support the treatment of, among others, renal failure, arthritis, or cognitive impairment syndrome. The amount of tryptophan in the diet has a great influence on the dog’s behavior, as this amino acid influences the activity of serotonin [14,73]. Studies in humans have long pointed to the influence of diet on behavior. A study conducted on children by Schoenthaler and Bier [69] found that a diet rich in vitamins and minerals can reduce antisocial behavior in school-aged children. Additionally, studies by Gesch et al. [73] confirmed these findings but also showed that supplementation with both vitamin and mineral supplements and essential fatty acids (SFA) such as docosahexaenoic acid—22: 6n-3 (DHA) reduced the occurrence of violence-related behaviors [73,74]. In the case of dogs, models of their behavior are also considered as a model for humans, and previous research indicates that diet plays an important role in the behavior of these animals [74].

For many years, the relationship between the amount of protein in the diet, including tryptophan metabolism, and aggressive behavior in dogs has been the subject of discussion and interest. Research by DeNapoli et al. [75] and Dodman et al. [76] showed that the level of protein in the dog’s diet is an important aspect in the treatment of canine behavioral disorders, particularly aggression. These studies were based on the use of two types of diets: high protein (HP) and low protein (LP) in dog nutrition. The use of an LP diet in combination with the addition of L-tryptophan (Trp) may be effective in reducing dominant aggression in dogs. The effect obtained with a low-protein diet may be related to obtaining an appropriate ratio of Trp to other large neutral amino acids (LNAA) in the blood plasma. In addition, a low-protein diet rich in carbohydrates may adversely affect the ratio of Trp and LNAA, similar to an HP diet. The imbalance between the above-mentioned amino acids may result in their increased competition for blood-brain transfer. Disruption of Trp transfer from the blood-brain pathway may reduce the production of serotonin (5-HT), which in turn may lead to impulsive aggression. L-tryptophan is a biosynthetic precursor of the neurotransmitter serotonin, as well as melatonin and niacin (B_3_). Therefore, it plays a key role in the diet of dogs and prevents the occurrence of, inter alia, impulsive aggression [77,78,79,80,81,82]. In addition, long-term supplementation at a dose of 1 g/dog per day resulted in an increase in voluntary food consumption, which also indicates its role in influencing the hormones involved in food intake and satiety regulation, such as leptin or cholecystokinin [82].

Another important aspect of the diet is the level of lipids, in particular the number of fatty acids such as eicosapentaenoic acid (EPA), conjugated linoleic acid (CLA), or docosahexaenoic acid (DHA). As mentioned earlier, human studies have found a link between dietary DHA levels and aggressive behavior. Lipids perform many key functions, including they are components of cell membranes, precursors of chemical messengers, and are used as a source of energy or stored in adipose tissue. Additionally, they are highly concentrated in the central nervous system. Additionally, a component of the brain’s gray matter is derived from dietary CLA (18: 2n-6) and α-linolenic acid (18: 3n-3). As a result of an enzymatic reaction, linolenic acid can be converted in the body into arachidonic acid and then into docosapentaenoic acid (22: 5n-6). In contrast, α-linolenic acid gives EPA (20: 5n-3), which can then be metabolized to DHA (22: 6n-3) [83,84,85]. Brain membrane phospholipids contain high levels of polyunsaturated fatty acids (PUFA), especially arachidonic and docosahexanoic acids [85]. Dietary PUFA levels affect cognitive functions in mammals, including dogs [86,87]. Combinations of DHA and EPA in a study by Kidd et al. [86] showed that they have a significant impact on various psychological functions, such as protection against hyperactivity and aggression. In addition, many studies in mammals (mainly mice) suggest that an altered lipid profile may be responsible for the occurrence of aggression and impulsiveness in animals [87]. A study by Re et al. [84] on German Shepherds showed that low cholesterol, bilirubin, docosahexaenoic acid, and a higher omega-6/omega-3 ratio might be correlated with the occurrence of aggressiveness in the tested dogs. Confirmation of the obtained results by Re et al. [84] can also be found in Sentürk and Yalçin [85], where the lipid levels of aggressive dogs were found to be lower than those of non-aggressive dogs. Both studies suggest that this was associated with lowering cholesterol and triglycerides and HDL–C, which negatively affects serotonin reuptake but requires further investigation.

Another important aspect of nutrition is the ability to influence the microbiome of the digestive system. Recent studies link the diversity of the composition of the gut microbiome with behavioral and psychological regulation in other mammals such as mice and humans, including aggressive behavior [88,89,90,91]. A study by Kirchoff et al. [91] on dogs of the pit bull type showed a correlation between the composition of the digestive system microbiome and the incidence of aggression in dogs. The authors of this study suggest the possibility of using specific probiotics in the future to stabilize the microbiome of aggressive dogs because, according to preliminary analyzes, the microbiome of aggressive dogs are characterized by a higher level of Firmicutes, Fusobacteria, Bacteroidetes, Proteobacteria phyla, including *Lactobacillus*, and a lower level of Bacteroides, however, it requires for further research [92,93]. In addition to probiotics, it is possible to administer substances of plant origin that may positively affect the microbiome of the digestive system, such as proanthocyanins derived from grapes. In a study by Scarsella et al. [93] where the aforementioned substance was used, it was shown that it affects the microflora of the digestive system in dogs, as well as the neuroendocrine response—an increase in serotonin levels after 28 days of using the supplement. The use of polyphenols in dog nutrition as a way of manipulation of the microbiome is associated with the supposition that intestinal microorganisms may be involved in the absorption of the polyphenolic structure due to the transformation into low molecular weight compounds [94,95]. However, this topic requires further research in dogs. 

However, it should be considered that most of the presented studies were carried out on a small number of individuals, which requires further research. 

## 4. Hormones and Aggressive Behavior

The biological basis of the aggression mechanism in the case of a domestic dog is still not fully understood, but there are many indications that hormones such as testosterone, estrone, or serotonin may play a role in it [96,97,98], As indicated in the available literature, in many cases the blame for the aggressive behavior of both male and female dogs is the sex hormones found naturally in their bodies [99]. In such a case, it is often recommended to castrate or sterilize aggressive individuals in order to reduce the existing level of aggression or other behaviors perceived as negative by the owners [100,101].

As performed studies show, the percentage of sterilized or castrated dogs is still growing, reaching almost 80–90% in many developed countries [102,103]. These treatments can bring many health benefits in terms of individuals and well-being for the population, but an increasing number of researchers indicate that lowering the level of sex hormones does not significantly reduce the number of negative behaviors or the level of individual aggression In the case of males, masculinization of the brain takes place during fetal life, and lowering testosterone levels in an adult or adolescent individual may not weaken these behaviors as evidenced by studies performed by McGreevy et al. [98]. Similar observations regarding the spaying procedure on females were presented by Starling et al. [99]. On the other hand, Farhoody et al. [104] showed the opposite phenomenon, wherein dogs subjected to gonadectomy there was a slight but statistically significant increase in the probability of aggression in relation to unknown persons, without in any way affecting the risk of aggression towards people and dogs they know.

In the available literature, we can also find publications indicating a relationship between the presence of low levels of serotonin, also commonly known as the happiness hormone, in the blood serum of dogs and the occurrence of impulsive aggression. In the case of the research conducted by Amat et al. [102], they showed that the English Cocker Spaniel is the breed particularly vulnerable to aggression on this basis. The serotonin level when comparing the ECS with aggressive dogs of other breeds was statistically significantly lower (*p* < 0.001) and amounted to 318.6 ± 67.1 vs. 852.77 ± 100.58 ng/mL. Similar conclusions related to the general mechanism of aggression in dogs were reached by the team of Rosado et al. [103], where they also noticed a statistically significant relationship between low serotonin concentrations and the occurrence of aggressive behavior in dogs. Dogs showing defensive aggression were characterized by the lowest observed concentrations of serotonin compared to non-aggressive dogs. They also observed that the aggressive dogs had significantly higher serum cortisol levels than the non-aggressive dogs. They noticed a statistically significant difference in dehydroepiandrosterone (DHEA) levels (respectively 90.9 ng/mL vs. 29.8 ng/mL) and the DHEA/cortisol ratio between males and females (respectively 9.5 vs. 3.8). 

MacLean et al. [105] indicate in their research the important role that the following hormones may play in regulating the mechanism of aggression in dogs, as in other mammals: vasopressin and oxytocin. In their two-stage studies, they showed that among service dogs that showed greater aggression in relation to the stranger who posed a threat, they had higher concentrations of vasopressin in the blood serum. However, it should be taken into account that most of the presented studies were carried out on a small number of individuals, which requires further research. 

The divergent positions scientists take regarding the hormonal foundations of aggression in dogs, and the not entirely clear biological basis of these behaviors cause a specific vacuum, which often negatively affects the welfare of dogs kept by humans. In the available literature, we can observe assumptions [106]. Concerning the existence of synergistic or antagonistic relations between the endocrine system and other biological systems, which may lead to the occurrence or enhancement of behaviors described as negative or aggressive. All these circumstances indicate a strong need for further research aimed at filling the gaps in our knowledge regarding the complex basis of aggressive behavior in dogs. The knowledge obtained in this way may contribute to the improvement of the welfare of dogs defined as aggressive and the potential reduction of the number of attacks by dogs on humans.

## 5. Neurocognitive Science in Canine Aggression

The science that is the effect of the combination of brain anatomy and experimental psychology is called neurocognitive science. According to neurocognitive scientists, a full understanding of the anatomy of both the human and animals brains will also allow us to more thoroughly explore behavioral sciences [107,108]. Biological psychology, neurocognitive science, and, consequently, modern brain imaging methods have also made it possible to learn more about the dog brain. Furthermore, it has been possible to identify certain similarities and differences between the canine and human brains [109].

In 2012, a series of fMRI studies began at Dog Star Technologies, Georgia, United States. It is an organization that deals with canine neuroscience, which goal is to understand dogs’ emotions, senses, and inhibitory control. The dogs’ responses to rewards were studied using magnetic resonance imaging. It found that 13 out of 15 dogs displayed similar brain activity for praise and food, which was the second variant of reward [109].

Research has shown that dogs, such as humans, have an area in the brain that processes faces. The area responsible for a dog’s vision was found to be strongly activated by faces. It has also been shown that dogs have the ability to recognize individuals of their own species from others, to distinguish pictures of landscapes from other dogs, to distinguish pictures of a human with a smiling face from one without emotion, and show different behavior at the sight of a picture of a dog and a person. These reactions were investigated using electroencephalography. It was usually invasive and required the use of a needle electrode. Advances in EEG have led to the use of adhesive electrodes, making it another promising and non-invasive method for imaging the brain of animals. As a result, animals do not need to undergo anesthesia, and their cognitive functions are not impaired and do not limit the performance of tests. The results of the examined reactions to visual stimuli were compared with the results of human studies. The visual evoked potential appeared faster in the dog than in the human because the human brain has more neurons and synapses than the dog’s brain, and, therefore, there are longer transmission delays. Moreover, components of the test also contribute to delaying the response to a visual stimulus. During the EEG, dogs looked at the displayed images passively without any additional tasks. On the other hand, the people taking part in the study were given tasks related to attention or memory. These variables affect the rate of transmission of the stimulus [102]. It is also worth mentioning the use of a non-invasive and helpful EEG test in the diagnosis of functional disorders of the central nervous system. It is great for the diagnosis of epilepsy—also in dogs. Dog brain imaging methods also allowed for a closer look at the phenomenon of aggression. The fMRI study discussed earlier showed a neurobiological response in dogs to seeing their owner feeding an artificial dog image. The second variant in the study was the observation of the owner putting food into the basket. The amygdala activation in both cases was compared with the results of the Canine Behavioral Assessment and Research Questionnaire (C-BARQ test). This test is a widely implemented instrument to evaluate dog behavior proven to be useful across various cultures [110]. The results clearly indicated that the dogs were more agitated and aggressive when observing their handler feeding the likeness of another dog. This mechanism resembles human jealousy. When the dog is exposed to such stimuli repeatedly, the amygdala gets used to the increased activity, and its intensity gradually decreases during the fMRI test. This phenomenon can be mainly observed in more aggressive dogs [111,112,113].

Aggression in both humans and other higher mammals is associated with changes in the structure of the brain [114,115,116,117,118]. In this type of research, two forms of aggression are distinguished: reactive aggression evoked in response to frustration/threat and instrumental aggression directed at the goal. It also states that there are different forms of the neurocognitive model, which in turn are necessary to explain the emergence of these different forms of aggression. Disturbances in executive emotional systems (somatoform system or social reversal system) are associated with the reactive aggression shown by patients with “acquired sociopathy” due to damage to the orbitofrontal cortex. The impairment of the ability to create associations between un-conditioned emotional stimuli, especially distress stimuli, and conditional stimuli (model of the violence inhibition mechanism) is associated with instrumental aggression of people with developmental psychopathy [107]. Studies focusing on the brain activation patterns by PET, SPECT, and fMRi have documented focal declines in the activity of the frontal and temporal cortex associated with various neuropsychiatric disorders. In single-photon emission computed tomography (SPECT), it was found that reduced prefrontal regional blood flow in the brain in patients with aggressive schizophrenia. The neuroimaging literature on aggression in mental disorders implies dysfunction in the frontotemporal circuits. These findings are consistent with the role of the medial temporal and ocular regions in emotional processing and executive cognition, which include abilities such as attention, planning, organization, abstract reasoning, self-control, and the ability to use feedback to modulate behavior. It is speculated that this leads to cognitive biases that increase the chances of aggressive behavior in response to stressful and provocative situations [116,117,118,119]. It was also shown that the volumes of the anterior cingulate cortex (ACC), orbitofrontal cortex (OFC), and amygdala were found to be significantly lower in aggressive individuals compared to healthy controls [120].

There is increasing evidence that specific neurobiological responses correlate with canine temperament and can help predict a dog’s future behavior. Exposure of more aggressive dogs to their owners’ interactions with other dogs, according to researchers, may find application in behavioral therapy. Such action may also be helpful for dogs whose aggression manifests itself in other cases [110]. There are many prejudices against dogs considered aggressive, but little knowledge about the neurological basis of aggressive behavior. In more aggressive dogs, in some situations, the excitement may be increased even without displaying specific impulsive behavior. The amygdala, which is part of the limbic system, is responsible for regulating many behaviors, including aggression. Any damage to the amygdala may result in aggression. Cancer, vascular disease, hormonal disorders, or another type of neurological trauma may contribute to this [117,118]. Aggression in dogs can also arise from a desire to defend its resources, including a handler to whom the dog wishes to restrict access. This can manifest itself in a variety of ways: from drawing attention to themselves, i.e., disturbing the handler with another person or dog, barking or growling. Another way is through aggressive behavior and attack. These observations became a reason to think that some manifestations of aggression can be equated with jealousy, which in dogs can occur as often as the more primal behaviors of fear and anger. One study found an aggressive reaction and behavioral arousal in dogs that watched their handlers interact with another dog. This observation was defined as the prototype of jealousy in dogs. Of course, despite these reports, there is no evidence to support the existence of jealousy in dogs. However, the cognitive abilities of dogs are highly developed. For example, they are good at using cues and are sensitive to social inequalities, for example, when one dog is rewarded and the rest is not [119].

However, for the test to be successful and accurate results, dogs need to be calm and relaxed, and such conditions can distract and upset him. For this purpose, 41 dogs were trained for eyeglass examination and 24 dogs for fMRi. The comprehensive training program included systematically accustoming the dog to a potentially stressful environment and desensitizing it to various stimuli, shaping and combining the required behaviors using reinforcements. For fMRi, dogs must be accustomed to 96 dB noise, vibration, and confined space through the scanner housing. The first step in training is getting your dog accustomed to entering the machine and keeping his head on the support. Then the presentation of a white image on the computer screen, which accustoms the dog to devices and the screen, which is 1 m from his eyes. The last stage is the calibration and verification of the eye movement tracking software with the monitor. Animation and moving images were used to attract the dog’s attention and keep it longer [120,121,122,123].

Learning more about neurocognition in dogs, however, requires further research but also allows us to broaden our knowledge of the occurrence of aggression and the methods that can be used to eliminate it for the benefit of the animal and its owners.

## 6. Conclusions

Studies from recent years related to negative dog behavior, especially aggression in dogs, and in particular its causes, have not been fully understood to this day. Recent studies show that factors such as disease, hormone balance, and diet may be associated with adverse aggressive behavior in dogs. However, new research also suggests that it is possible to correct or avoid the occurrence of undesirable aggressive behavior in dogs by correctly reading dogs’ body language, understanding their basic needs, adjusting their diet to training, identifying disease early, introducing proper socialization, and understanding that aggression is a natural part of canine ethogram. It is also connected with the necessity of educating future dog owners or developing diagnostic apparatus/methods. A forward-looking understanding of all aspects of aggression, from physiology to environment, can help eliminate adverse behavioral changes in dogs and thus improve their welfare, which is crucial for the animals themselves.

## Figures and Tables

**Table 1:** Selected aggression behavior definitions.

**DEFENSIVE AGGRESSION**
*Occurs when the dog, due to the lack of socialization with other animals, reads other animals as deadly, painful procedures or their arrival are associated with pain and irritation, the safety limit for a bitch protecting her young is exceeded, as well as a safety limit for a dog protecting his area* [16]
**DISTANCING AGGRESSION**
*This is a symptom of social anxiety disorder. Through distancing aggression, the dog manifests pathological fear or anxiety in contact with other animals or dogs* [17]
**TERRITORIAL AGGRESSION**
*The goal of territorial aggression is to keep the threat at a safe distance. Defending a territory is a natural behavior for a dog, which is why dog owners praise those who warn against intruders and guard the property, which makes the problem of territorial aggression worsen in every situation of this type. Aggression towards postmen is the biggest factor that deepens this type of aggression due to the fact that the systematic appearance of the postman and the reward by the dog, which is the departure of the postman, strengthens this type of behaviors* [9].
**MATERNITY AGGRESSION**
*It is a type of defensive aggression of varying severity. Its aim is to chase away intruders who could threaten the puppies or injure the bitch, which may have a negative impact on the further rearing of the young. When the intruder is a stranger, the attack is brutal and direct. Maternal aggression resembles territorial and distancing aggression, because the female dog in most cases, fiercely defends not only the puppies but also the place where she gave birth or the place where the puppies usually live* [18]
**AGGRESSION FROM DISEASE**
*It is a type of defensive aggression of moderate intensity. This type of aggression is characteristic of dogs forced to do something, hurt or upset. A common stimulus is an illness or wound that requires care. Unfortunately, usually in such situations the dog’s guardian intervenes, who may become a victim of aggression, which may deteriorate the bond between him and the dog* [19]
**AGGRESSION OUT OF FEAR**
*It is a type of defensive aggression that is a natural behavior for any living being. This type of aggression caused by fear should be treated as an incident, not a habit, but it should be remembered that this type occurs during anxiety disorders, the basis of which should be found and eliminated* [20]
**DISPLACED AGGRESSION**
*It is aggression that can arise from any other type of aggression. A distinctive feature is a high agitation. In this case, the dog tries at all costs to transfer its agitation and aggression to the object closest to it. This type of aggression is especially unpredictable because a dog that is aggressive towards another animal may seek to vent its aggression by attacking its handler, who is within reach of his jaws. Displaced aggression is an automatic behavior that can be found in explosive and impulsive individuals* [17]
**COMPETITIVE AGGRESSION BETWEEN DOGS**
*This type of aggression is perceived as a game in which the participants, depending on their social positions, try to impress the opponent by adopting various poses. It should not end in serious injuries. The conflict allows you to verify which side is dominant and which side is defeated* [15]
**COMPETITIVE AGGRESSION BETWEEN DOGS AND PEOPLE**
*As in the case of competitive aggression between dogs, this time, it is a kind of game. Unfortunately, this time the man from above is in a losing position because he cannot take part in this game, which in turn may result in bodily harm. Not knowing how to read this type of aggression contributes to a wrong assessment of the situation by a human and rewarding the dog for dominating the owner, about which the dominated person himself has no idea* [20]
**POSSESIVE AGGRESSION**
*It occurs when the dog defends access to its own or stolen property, person, land, or food. It happens that by provoking the owner, the dog verifies its position in the hierarchy or tries to convince him to play* [13]
**AGGRESSION BETWEEN DOGS BELONGING TO DIFFERENT SOCIAL GROUPS**
*This type of aggression is used when dogs from different social groups meet outside their territories and have a strong need to establish a hierarchical position among themselves* [15]
**HUNTING AGGRESSION**
*Hunting aggression does not end with killing the victim. This type of aggression can be eliminated by properly socializing the dog with objects or situations such as runners or cyclists. As a predator, the dog reacts to moving objects or creatures by chasing, which is a natural behavior for him. As in the case of hunting aggression, there are breeds whose tendencies to chase are conditioned by genes and its utility* [18]
**HUNTER AGGRESSION**
*The case of this type of aggression is unique in that the behavior and the dog’s facial expressions are unlike any other type of aggression. The dog’s muzzle remains smooth, and the dog only becomes emotionally aroused in a manner characteristic only of hunting. As a predator, the dog can hunt anything that moves that has not been socialized with. The victim may be a creature or thing that the dog considers edible or not representative of its species. This also applies to small breeds of dogs when the aggressor is a representative of a large breed of dog. The occurrence of hunting aggression is largely determined by the genes and utility of the breed* [18]
**EXCESSIVE AGGRESSION**
*Excessive aggression is divided into secondary, primary, and command. In the case of excessive secondary aggression, it gradually transforms from other types of aggression, while in the case of primary excessive aggression, it appears suddenly. In both cases, we are dealing with pathology, and the tendency to these types of aggression may indicate an explosive personality or diseases such as brain tumors or schizophrenia. Behavior in both cases of excessive aggression is diagnosed as illogical and non-functional* [21,22]

## Data Availability

Not applicable.
